# Black Wheat Extracts (Arriheuk) Regulate Adipogenesis and Lipolysis via Adenosine Monophosphate (AMP) Activated Protein Kinase (AMPK)/Sirtuin 1 (SIRT1) Signaling Pathways

**DOI:** 10.3390/foods12142727

**Published:** 2023-07-18

**Authors:** Young Yoon, Min-Kyung Park, Kyung-Hoon Kim, Geum-Hwa Lee

**Affiliations:** 1 Imsil Cheese & Food Research Institute, Doin 2-gil, Seongsu-myeon, Imsil-gun 55918, Republic of Korea; kuburi79@icf.re.kr (Y.Y.); m930916@icf.re.kr (M.-K.P.); 2 National Institute of Crop Science, Rural Development Administration, Wanju 55365, Republic of Korea; k2h0331@korea.kr; 3 Non-Clinical Evaluation Center, Biomedical Research Institute, Jeonbuk National University Hospital, Jeonjusi 54907, Republic of Korea

**Keywords:** arriheuk, wheat, flavonoids, 3T3-L1 adipocytes, PGC-1α, AMPK, SIRT1

## Abstract

Polyphenols and other compounds with antioxidant properties are found in plants and are one of the main antioxidants proven to reduce body weight and the risk of insulin resistance. Still, the mechanism behind the protective effects against obesity remains unclear. Thus, the study aims to assess the impact of flavonoid-rich arriheuk extract, a purple wheat extract, on mitochondrial function using 3T3-L1 adipocytes and investigate the molecular mechanism behind its protective effects against adipogenesis and lipolysis. The study findings strongly indicate that arriheuk significantly suppressed triglyceride levels and inhibited the expression of transcription factors like C/EBPα and PPARγ in 3T3-L1 adipocytes. Furthermore, treatment with arriheuk suppressed the expression of SREBP1c and FAS proteins linked to lipogenesis. In addition, treatment with arriheuk extract decreased the mRNA levels of adipogenic transcription factors, increased glycerol release, and inhibited adipocyte differentiation. Interestingly, the arriheuk-mediated PGC-1α expression triggered mitochondrial biogenesis by promoting the AMPK phosphorylation and SIRT1 expression in adipocytes. Also, arriheuk suppressed adipogenesis and elicited browning through the AMPK- and SIRT1-associated pathways. Collectively, these findings strongly suggest that arriheuk extract regulates browning in 3T3-L1 white adipocytes by triggering the AMPK/SIRT1 pathway, indicating the prospective applications of arriheuk as a functional food to control obesity.

## 1. Introduction

Obesity is a complicated condition caused by an unbalanced energy metabolism due to excessive fat accumulation that induces multiple health risks, including diabetes mellitus, hypertension, and cardiovascular diseases [1,2,3]. Thus, obesity is one of the most prominent health issues in our modern society. Based on the National Health and Nutrition Examination Survey, the risk of hypertension, diabetes, metabolic disease, and cancer is more than two-fold among obese individuals. Obesity and its associated metabolic disorders have significantly increased throughout the world, contributing to morbidity and mortality. Adipocytes exhibit characteristics that influence the homeostasis of the metabolism, which is a potential therapeutic target for obesity. It is well known that white adipose tissue (WAT) is ideally designed to store energy as triglycerides (TGs) [3,4,5]. In addition, lipogenesis and lipolysis modulate the buildup of lipids in WAT, a process that involves transcription factors, proteins, and enzymes. Moreover, AMP-activated protein kinase (AMPK) and sirtuin 1 (SIRT1) are considered fuel-sensing enzymes [6,7], while transcription factors like CCAAT enhancer-binding protein alpha (C/EBPα), sterol regulatory element-binding protein 1c (SREBP-1c), and peroxide proliferative activated receptor gamma (PPARγ) are known to regulate differentiation and control several downstream genes involved in the lipid metabolism [8]. PGC-1 is regarded as a master regulator owing to its involvement in thermogenesis at the molecular level and as a transcription coactivator, which makes it essential for the differentiation of brown adipocytes [9]. Thus, AMPK, SIRT1, and PGC-1 established a network for energy sensing that regulates mitochondrial energy expenditure. Therefore, obesity is linked to the disruption of the AMPK/SIRT1 pathway, which potentially plays a vital role in the development of obesity. Therefore, natural products that promote lipolysis or restrict fat formation with fewer adverse effects are needed for managing obesity.

Wheat (Triticum aestivum) is a widely cultivated staple crop used in a variety of cuisines. Wheat bran, a byproduct of wheat processing, typically represents up to 20% of the grain’s weight and up to 55% of total dietary fiber [10,11,12]. Furthermore, polyphenols present in wheat have antioxidant and anti-inflammatory properties [13,14]. Interestingly, flavonoids are abundant in the aleurone layers of purple-colored wheat and confer a significant nutritional advantage over common wheat [5,14,15,16,17]. There is a great deal of interest in applying germination processes that can substantially improve nutritional and health benefits of grains by increasing the amount of bioactive phytochemicals in the sprouts [15]. Moderately hydrophobic polyphenolic fractions and hydrophilic peptidic fractions were found in wheat sprouts [16]. The presence of a total phenolic compound content is correlated with high antioxidant activity [17]. Moreover, the high antioxidant capacity of wheat sprouts can support their use as food supplements to act against diseases induced by free radicals [18]. ‘Arriheuk’ is a purple colored wheat variety developed by the National Institute of Food Science, Wanju, of the Republic of Korea, under the breeding program to promote the production and consumption of domestic wheat [18]. There is currently great interest in the application of germination processes that can significantly enhance the dietary and health benefits of grains by increasing the content of bioactive phytochemicals in the sprouts [15]. Moderately hydrophobic polyphenolic fractions and hydrophilic peptidic fractions were found in wheat sprouts [16]. The presence of the total phenolic compound content is correlated with high antioxidant activity [17]. The high antioxidant capacity of wheat sprouts can support their use as food supplements to act against the diseases induced by free radicals [18]. The nutritional benefits of this purple wheat are of interest to health enthusiasts due to its high levels of polyphenolic compounds. The flavonoids in the arriheuk enhance metabolic control in obesity by lowering lipogenesis, oxidative stress, and inflammation [19]. These beneficial flavonoids are primarily derived from the wheat hull (primarily bran) and aleurone layer. This study aims to assess the anti-adipogenic effect of the arriheuk extracts in 3T3-L1 adipocytes and to investigate the potential regulatory mechanisms with a special focus on AMPK/SIRT1 signaling.

## 2. Materials and Methods

### 2.1. Extraction and Purification of Purple Wheat (Arriheuk)

Purple wheat (arriheuk) used in this study was provided by the National Institute of Food Science, Wanju, Republic of Korea. The black wheat was finely powdered, dried, and extracted with either hot water or ethanol for general applications. Specifically, the aleurone layer was removed from the wheat and extracted with the same amount of hot water/50% ethanol/70% ethanol at room temperature (18 ± 2 °C) for 12 h. This process was repeated twice. The resultant solvent was filtered, concentrated at 60 °C under 100 hPa pressure, and lyophilized using a freeze dryer. The yield was determined by dividing the weight of the lyophilized powder by the weight of the raw material. The total flavonoid content (TFC) of the arriheuk extracts were determined by high-performance liquid chromatography (HPLC) as suggested in the previous study with minor modifications in Table 1 [18].

### 2.2. 3T3-L1 Cell Culture and Differentiation

The culture of 3T3-L1 preadipocytes was performed as previously reported [20]. Briefly, 3T3-L1 preadipocytes were cultured for 48 h in DMEM with fetal bovine serum (FBS) and 1% penicillin/streptomycin. Regarding the differentiation of adipocytes, preadipocytes were induced by adding 1 µg/mL insulin, 1 mM dexamethasone, and 0.5 mM 3-isobutyl-1-methylxanthine and co-treated with 2, 10, or 50 µg/mL of arriheuk (WEA, 50EEA, and 70EEA) for 3 days. The induction medium was changed with 1 µg/mL insulin for another 5 days, and a fresh medium was changed every 2 days. Cells were cultured under a controlled humidified atmosphere at 37 °C with 5% CO_2_.

### 2.3. MTT Assay

The MTT assay was carried out to assess the metabolic activity of 3T3-L1 cells. 3T3-L1 cells (1 × 10^5^ cells/well) were cultured for 24 h. Next, the culture medium was substituted with a culture medium having 100 µg/mL arriheuk extracts. After 24 h, the cells were incubated at 37 °C for 4 h with 10 µL of MTT solution (Sigma, St. Louis, MO, USA). Absorbance was measured using absorbance at 590 nm using a multiwell plate reader.

### 2.4. Oil Red O Stain

Oil Red O staining was employed to determine the accumulation of intracellular lipids and performed as described previously [20]. The cultured cells were treated with wheat extracts for 24 h. After 24 h, cells were rinsed with PBS, fixed, and stained with Oil Red O solution at room temperature. Later, the cells were treated with isopropyl alcohol to get rid of unbound dye, and Oil Red O spectra were measured at 490 nm using a Fluoroskan^TM^ FL microplate reader (Thermo Fisher Scientific, Waltham, MA, USA).

### 2.5. Immunoblotting Analysis

Immunoblotting was performed as previously described [21]. Homogenates of 3T3-L1 cells were separated using MA, USA), p-HSL (#45804, Cell Signaling, MA, USA), HSL (#4107, Cell Signaling), UCP-1 (#sc-293418, Santa Cruz, CA, USA), PGC-1α (#sc-518025, Santa Cruz), SIRT1 (#8469, Cell Signaling), AMPK (#5832, Cell Signaling), and p-AMPK (#2535, Cell Signaling). All the band intensities were quantified with ImageJ (NIH, Bethesda, MD, USA).

### 2.6. Immunofluorescence

Immunofluorescence was performed as suggested in the previous reports [20]. The differentiated adipocytes were fixed, washed, and permeabilized with 0.1% Triton X-100. The cells were incubated overnight at 4 °C against UCP1 (#sc-293418, Santa Cruz). Then, the cells were labeled with a fluorescence-conjugated anti-mouse IgG-TRITC (#A16071, Thermo Fisher Scientific). Then, the nuclear counterstaining was done with Hoechst 33342 (#H3570, Thermo Fisher Scientific). Finally, the cells were mounted and imaged with the EVOS M5000 Cell Imaging System (Thermo Fisher Scientific).

### 2.7. Nile Red Staining

Neutral lipid staining in mature adipocyte cells was carried out as suggested in previous investigations [20]. Briefly, differentiated 3T3-L1 cells were stained with 0.5 μg/mL Nile red solution (#N1142, Thermo Fisher Scientific) for 10–15 min at room temperature. Then, cells were thoroughly washed and counterstained with 4,6-diamidino-2-phenylindole (DAPI). Next, cells were mounted and imaged with EVOS M5000 Cell Imaging System (Thermo Fisher Scientific). 

### 2.8. Determination of Adipogenesis and Lipolysis in 3T3-L1 Cells

The regulation of energy balance is dependent upon lipolysis. Triglycerides (TGs) are hydrolyzed during lipolysis to produce glycerol and free fatty acids (FFAs). Thus, to determine the extent of lipolysis, the free glycerol, FFA, and TG concentrations in the cell culture medium were measured. Glycerol was measured using Glycerol Cell-Based Assay kit (#K977-100, BioVision, Milpitas, CA, USA). Isoproterenol (#SLBK3425V, Sigma) was used as a positive control for the assay. TG concentration was measured with Triglyceride from BioVision. The results of the TG levels were expressed as % of triglycerides compared to control. Isoproterenol (#SLBK3425V, Sigma) was used as a positive control for the assay. FFA was assessed using FFA assay kit (K612-100, BioVision).

### 2.9. Gene Expression Analysis

Gene expression analysis was carried out as suggested in previous investigations [20]. Briefly, the total RNA was isolated with Trizol solution (#15596026, Invitrogen, Carlsbad, CA, USA), and cDNA was synthesized using the oliogo dT primer kit (#6110A, TaKaRa Bio Inc., Shiga, Japan). The primer sequences used for PCR in this investigation are listed in Table 2. qRT-PCR was carried out with SYBR^®^ Premix Ex Taq ™ master mix (#RR041A, TaKaRa Bio Inc.,) on an ABI PRISM 7500 Real-Time PCR system (Applied Biosystems, Foster City, CA, USA). For the quantification of PCR products, the Ct method was adopted. The level of β-actin expression was used to calibrate each sample.

### 2.10. Statistical Analysis

GraphPad Prism v8.0 (GraphPad Software, San Diego, CA, USA) was used to analyze the investigational data. For multiple comparisons, one-way ANOVA with Tukey’s post hoc test was performed. Data are expressed as the mean ± SEM. *p* < 0.05 indicates statistical significance. 

## 3. Results 

### 3.1. Cell Viability and Cytotoxicity of Arriheuks in 3T3-L1 Cells

**Figure 1:**
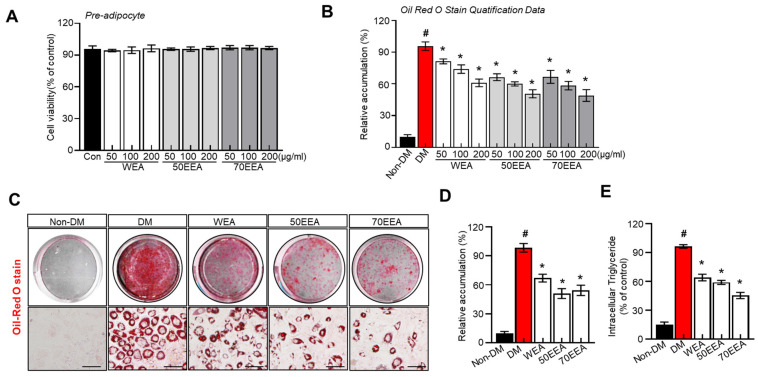
**Effect of arriheuk on cell proliferation and lipid accumulation in 3T3-L1 cells.** (**A**) 3T3-L1 cells were incubated with or without (50, 100, and 200 μg/mL, respectively) WEA, 50EEA, and 70EEA for 48 h. (**A**) Cell viability was evaluated by the MTT test. (**B**) Quantification of lipid accumulation. (**C**) Representative images showing Oil Red O-stained cells treated with 100 μg/mL arriheuk extracts extracted by different methods. (**D**) Quantification of lipid droplets of Oil Red O-stained cells (treated with 100 μg/mL arriheuk extracts). (**E**) Intracellular TG levels. Oil Red O-stained cells images were captured at 200× magnification. The scale bar indicates 50 μm. Data are presented as mean ± SEM. n = 3, ^#^ *p* < 0.05 vs. non-DM group, * *p* < 0.05 vs. DM group. DM, differentiated medium; non-DM, undifferentiated group; WEA, hot water purple wheat extract; 50EEA, 50% ethanol purple wheat extract; 70EEA, 70% ethanol purple wheat extract.

The cell viability remains similar in all the groups (Figure 1A). The fat accumulation in the groups treated with 50, 100, and 200 μg/mL of arriheuk hot water and ethanol extracts (50EEA and 70EEA) demonstrated dose-dependently reduced fat accumulation, which was lower than the control group (Figure 1B). These findings suggest that arriheuk produced from ethanol is of higher quality than arriheuk extracted from water, as the total flavonoid content of the ethanol extract is greater than the water extracts. Moreover, all the extracts markedly alleviate lipid accumulation on 3T3-L1 adipocytes, displaying the dose-dependent differentiation compared to differentiated cells (Figure 1C,D). Lipolysis involves the breakdown of intracellular TGs, making it the primary constituent of lipid droplets. In this study, arriheuk administration significantly decreased the intracellular TG content compared to other groups (Figure 1E). These significant findings suggest that arriheuk can effectively inhibit adipocyte differentiation and lipid accumulation.

### 3.2. The Effect of Arriheuk on Transcription Factors of Adipogenesis

**Figure 2:**
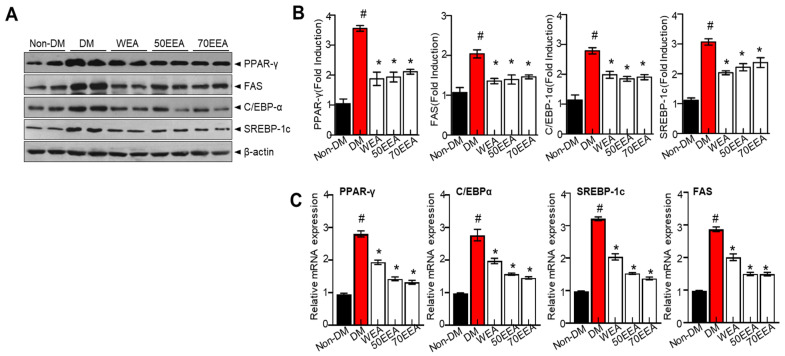
**Effects of arriheuk on the expression of adipogenic factors in 3T3-L1 cells.** Adipocyte differentiation was triggered with 100 μg/mL arriheuk. (**A**) Immunoblotting of PPARγ, FAS, C/EBPα, and SREBP-1c and (**B**) respective quantification analysis. (**C**) mRNA expression levels of PPARγ, C/EBPα, SREBP-1c, and FAS. Data are presented as mean ± SEM. n = 3, ^#^ *p* < 0.05 vs. non-DM group, * *p* < 0.05 vs. DM group. DM, differentiated medium; non-DM, undifferentiated group; WEA, hot water purple wheat extract; 50EEA, 50% ethanol purple wheat extract; 70 EEA, 70% ethanol purple wheat extract.

The expressions of adipogenesis factors were evaluated to determine whether arriheuk reduces adipocyte differentiation. PPAR-γ and C/EBPα are considered to be the primary transcription factors and are highly expressed in adipose tissue. Thus, the protein and gene expressions of PPARγ, C/EBPα, FAS, and SREBP-1c were analyzed to determine the effects of arriheuk on adipogenesis-related genes. Arriheuk treatment significantly reduced the expression of PPAR-γ, FAS, SREBP-1c, and C/EBPα proteins (Figure 2A,B). Similar observations were recorded with respect to the mRNA levels of PPAR-γ, FAS, SREBP-1c, and C/EBPα (Figure 2C).

### 3.3. Arriheuks Increase Glycerol Release and the Phosphorylation of HSL in Differentiated 3T3-L1 Adipocytes

**Figure 3:**
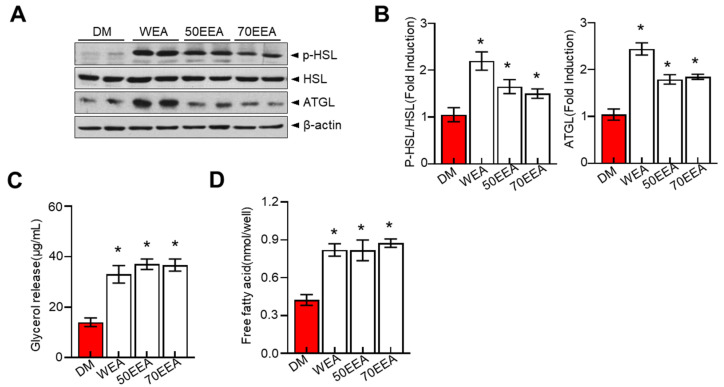
**Effects of arriheuk on lipolysis determined by measuring the glycerol release and the expression of ATGL, HSL, and pHSL in differentiated 3T3-L1 adipocytes.** Adipocyte differentiation was induced in the presence of 100 μg/mL arriheuk. (**A**) Protein expression levels of ATGL, p-HSL, and HSL were analyzed by immunoblotting and (**B**) quantification analysis. (**C**) Glycerol release concentrations in different groups. (**D**) Free fatty acid contents in different treatments. Data are presented as mean ± SEM. n = 3, * *p* < 0.05 vs. DM group. DM, differentiated medium; non-DM, undifferentiated group; WEA, hot water purple wheat extract; 50EEA, 50% ethanol purple wheat extract; 70EEA, 70% ethanol purple wheat extract.

Adipose triglyceride lipase (ATGL) and hormone-sensitive lipase (HSL) are the major determining enzymes for lipolysis in adipocytes. These proteins serve a crucial role in regulating fat accumulation and its breakdown. Therefore, the lipolytic effect of arriheuk on differentiated 3T3-L1 adipocytes was assessed based on its ability to induce glycerol levels in the culture medium and the intracellular phosphorylation of HSL and ATGL. In this study, arriheuk suppressed the levels of ATGL and p-HSL proteins (Figure 3A,B), while glycerol and free fatty acid (FFA) release were significantly increased (Figure 3C,D).

### 3.4. Arriheuk Regulates Mitochondria Biogenesis and Induces 3T3-L1 Browning

**Figure 4:**
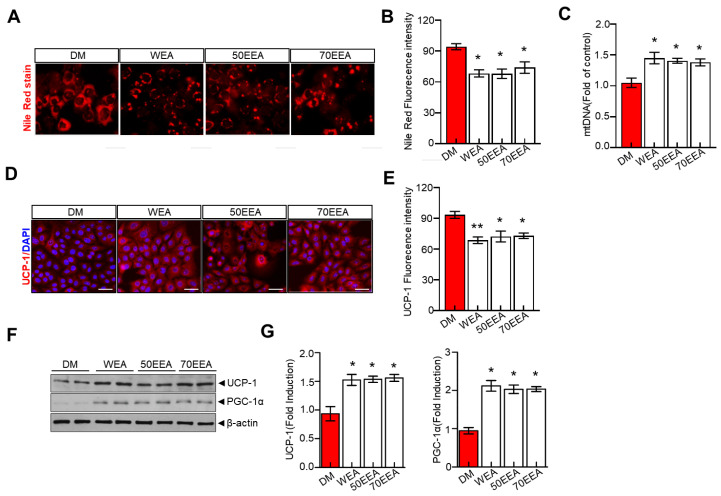
**Arriheuk promotes mitochondrial biogenesis in 3T3-L1 adipocytes cells.** Adipocyte differentiation was induced in the presence of 100 μg/mL arriheuk. (**A**) Representative images showing Nile red staining. All the images were acquired using a confocal microscope. Scale bar = 20 µm and (**B**) quantitative analysis of Nile red staining activity. (**C**) Quantification of adipocyte mtDNA normalized to total genomic DNA by qPCR. (**D**)Representative confocal micrographs of immunofluorescence staining of UCP1 (scale bar, 20 µm), and (**E**) quantification of staining intensity. (**F**) Immunoblotting of UCP-1 and PGC-1α and (**G**) quantify cation analysis of UCP-1 and PGC-1α. Data are presented as mean ± SEM. n = 3, * *p* < 0.05, ** *p* < 0.01 vs. DM group. DM, differentiated medium; WEA, hot water purple wheat extract; 50EEA, 50% ethanol purple wheat extract; 70EEA, 70% ethanol purple wheat extract.

All factors involved in the browning of fat were examined to understand the potential molecular mechanism underlying the arriheuk-induced browning of fat. Additionally, the Nile red staining was performed to visualize the fat accumulation. Mitochondrial DNA (mtDNA) was also evaluated as a proxy biomarker of mitochondrial health. Furthermore, cells were treated with an adipocyte differentiation cocktail and treated with arriheuk to measure brown fat markers such as PGC-1α and UCP-1. Here, treatment with arriheuk controlled the fat accumulation (Figure 4A) and improved the mitochondrial function indicated by the levels of mtDNA (Figure 4B). Moreover, the arriheuk treatment upregulated the expression levels of PGC-1α and UCP1 in 3T3-L1 adipocytes (Figure 4D–G).

### 3.5. Effect of Arriheuk on the SIRT1–AMPK Axis in 3T3-L1 White Adipocytes

**Figure 5:**
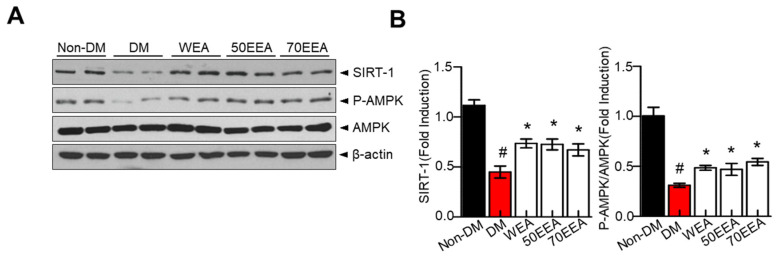
**The effects of arriheuk on the expression of SIRT1, p-AMPK, and AMPK in 3T3-L1 adipocytes.** Adipocyte differentiation was induced in the presence of 100 μg/mL arriheuk. Differentiated 3T3-L1 cells were treated with WEA, 50EEA, and 70EEA. (**A**) Immunoblotting of SIRT1, AMPK, and p-AMPK. (**B**) Quantification of SIRT1 and p-AMPK/AMPK ratio. Data are presented as a mean ± SEM. n = 3, ^#^ *p* < 0.05 vs. non-DM group, * *p* < 0.05 vs. DM group. DM, differentiated medium; non-DM, undifferentiated group; WEA, hot water purple wheat extract; 50EEA, 50% ethanol purple wheat extract; 70EEA, 70% ethanol purple wheat extract.

AMPK restores the energy balance during metabolic stress [6] and suppresses adipocyte differentiation during differentiation. Thus, we investigated whether arriheuk activates the metabolic shift-associated SIRT1–AMPK signaling axis in 3T3-L1 adipocytes. Treatment with arriheuk recovered SIRT1 and p-AMPK protein expressions and SIRT1 activity in differentiated 3T3-L1 cells (Figure 5A). Moreover, the p-AMPK/AMPK ratio was elevated with the arriheuk treatment (Figure 5B). These findings suggest that arriheuk extract inhibits adipocyte differentiation and lipolysis via the AMPK–SIRT1 axis.

## 4. Discussion

Antioxidants help in obesity management. The antioxidants and flavonoids present in the arriheuk extract potentially influence mitochondrial biogenesis, but the mechanism by which they prevent obesity is unclear. Arriheuk, purple wheat extract is a rich source of polyphenolic compounds. Hence, arriheuk consumption could benefit obesity. Thus, this study aims to investigate the anti-adipogenic effect of the extract of arriheuk (Purple wheat extract, PWE) in 3T3-L1 adipocytes and potential regulatory mechanisms with a special focus on AMPK/SIRT1 signaling.

Firstly, cytotoxicity assays reveal that arriheuk is non-toxic, as treatment with arriheuk did not alter cell viability. These observations show the potential use of arriheuk as a functional food to manage obesity. Here, the mitochondrial biogenesis and AMPK activation during 3T3-L1 adipocyte differentiation were evaluated to understand the effect of the underlying mechanism behind the positive implications of arriheuk. The findings strongly suggest that arriheuk positively affects lipogenesis and adipocyte differentiation via a metabolic shift that regulates fat accumulation. During 3T3-L1 adipocyte differentiation, arriheuk extracts significantly inhibited lipid accumulation by suppressing SREBP-1C, PPARγ, C/EBPα, and FAS expressions. Specifically, adipocyte differentiation necessitates SREBP-1c, which induces the expression of PPARγ and FAS expressions [22]. Furthermore, the expression of transcription factors like C/EBPα and PPARγ facilitates lipogenesis [23]. Also, these transcription factors are crucial to regulators that mediate the pathogenesis of metabolic diseases such as obesity [24]. Here, the suppression of the transcription factors with arriheuk is evident in 3T3-L1 adipocytes. Arriheuk in adipocytes is correlated with the expression of UCP-1 and PGC-1 proteins, supporting the observation that arriheuk decreases intracellular fat accumulation in adipocytes.

Furthermore, during the early phases of adipocyte differentiation, C/EBPβ and C/EBPδ are expressed and induce the expression of C/EBPα and PPARγ, which are the most significant modulators of adipogenesis. PPARγ is the critical regulator of adipocyte proliferation and differentiation [25]. Previous reports indicate that PPARγ-deficient cells cannot differentiate into mature adipocytes amid the ectopic expression of numerous pro-adipogenic factors [26]. Additionally, PGC-1α and UCP1 stimulate mitochondrial biogenesis and oxidative phosphorylation during cellular energy metabolism [27,28]. Thus, these genes are crucial biosynthetic markers for the replication and synthesis of mitochondrial DNA. Arriheuk elevated the expression levels of UCP-1, PGC-1α, and PPARγ in 3T3-L1 adipocytes.

AMPK, an important modulator of energy metabolism, influences the expression of key factors involved in mitochondrial biogenesis and energy expenditure in BAT, regulating lipid metabolism. The enhanced AMPK increases nicotinamide phosphoribosyltransferase (NAMPT), which helps to maintain fatty acid oxidation via mitochondrial respiration. Additionally, fatty acid oxidation increased the NAD^+^ levels [29]. Enhanced NAMPT and NAD^+^ trigger SIRT1 activity and stimulate PGC-1α deacetylation and activation [30]. Additionally, PGC-1 is activated by AMPK via either direct phosphorylation or by facilitating SIRT1-dependent deacetylation. Additionally, the ability of BAT to dissipate energy as heat suggests the anti-inflammatory actions of BAT, where UCP-1 regulates the release of heat [31], regulating thermogenesis. Collectively, PGC-1α functions as key regulator of mitochondrial biogenesis and influence UCP-1, which influences mitochondrial function by modulating energy expenditure, heat production, and ROS levels. This complex relationship between PGC-1, UCPs, and mitochondrial biogenesis is positively regulated by arriheuk in 3T3-L1 adipocytes to maintain cellular energy homeostasis, where physiological processes such as metabolism, thermogenesis, and oxidative stress response are regulated to exert beneficial effects. Previously, SIRT1 activated PGC-1 via deacetylation and AMPK, thereby directly mitochondrial DNA transcription [32]. Here, treatment with arriheuk significantly revived p-AMPK and SIRT1 protein expression levels.

Interestingly, caffeic acid (3,4-dihydroxycinnamic acid, CA) and its derivatives commonly occur in plant species, especially as major secondary metabolites in coffee and tea. Isovanillic acid (IA) is long recognized as beneficial in obesity and type 2 diabetes management. Also, polyphenols like CA and IA acid are known to inhibit lipid oxidation and ROS formation in vitro and in vivo. Evidence suggests that CA and IA induce non-shivering thermogenesis by primarily impacting adipogenic differentiation via AMPK activation. Here, the observations of the study strongly suggest a close connection between mitochondrial biogenesis and AMPK/SIRT1 activation. Also, it is indicated that the arriheuk-linked reduction in adipogenesis and mitochondrial biogenesis are directly linked to AMPK/SIRT1 activation. Thus, AMPK/SIRT1 is a likely therapeutic target for obesity and its metabolic effects. Together, the observations of the study confirmed that arriheuk regulates obesity by suppressing adipogenic factors and promoting thermogenesis involving AMPK–SIRT1–PGC-1.

### Limitations of This Study

The current investigation provides conclusive evidence on the underlying mechanism of arriheuk against adipocyte differentiation and obesity. However, further in vitro tests with AMPK/SIRT1 knockout technologies are necessary to validate the suggested mechanism. Additionally, similar in vivo tests are necessary to corroborate the investigation observations. These advanced in vitro and in vivo studies confirm the differentiation and browning of adipocytes through AMPK activation. Also, other compounds in the wheat extract apart from polyphenolic compounds may be involved in the beneficial effect and must be investigated to validate the contribution of polyphenolic compounds.

## 5. Conclusions

In summary, this study reports that arriheuk inhibited lipid and TG accumulation in 3T3-L1 adipocytes. Additionally, the beneficial effects of arriheuk can be attributed to the AMPK/SIRT1/PGC-1 signals that regulate adipocyte differentiation. Thus, arriheuk can be regarded as a functional food that can prevent mitochondrial dysfunction and adipocyte differentiation associated with obesity.

## Figures and Tables

**Table 1:** Flavonoids in the prepared arriheuk extract.

	Concentration of Flavonoids (μg/g)		
	Ethanol–Water (50–0)	Ethanol–Water (70–30)	Water
Gallic acid	648.6	407.0	479.7
Protocatechin	1343.2	640.5	528.7
Caffeic acid	2170.6	1284.4	890.9
Isovanillic acid	1567.4	1161.7	526.6
*p*-coumaric	377.4	267.3	112.8
Ferulic acid	478.9	275.4	388.0

**Table 2:** Real-time PCR primer sequences.

Gene	Sense (5′–3′)	Antisense (5′–3′)
SREBP-1c	CAAGGCCATCGACTACATCCG	CACCACTTCGGGTTTCATGC
PPARγ	CGCTGATGCACTGCCTATGA	AGAGGTCCACAGAGCTGATTCC
FAS	CTCATCCACTCAGGTTCAG	AGGTATGCTCGCTTCTCT
C/EBPα	CGCAAGAGCCGAGATAAAGC	CACGGCTCAGCTGTTCCA
β-actin	AAGACCTCTATGCCAACAC	CTGCTTGCTGATCCACAT

## Data Availability

Data is contained within the article.
