# Correction: Yoon et al. Black Wheat Extracts (Arriheuk) Regulate Adipogenesis and Lipolysis via Adenosine Monophosphate (AMP) Activated Protein Kinase (AMPK)/Sirtuin 1 (SIRT1) Signaling Pathways. *Foods* 2023, *12*, 2727

**DOI:** 10.3390/foods14162816

**Published:** 2025-08-14

**Authors:** Young Yoon, Min-Kyung Park, Kyung-Hoon Kim, Geum-Hwa Lee

**Affiliations:** 1 Imsil Cheese & Food Research Institute, Doin 2-gil, Seongsu-myeon, Imsil-gun 55918, Republic of Korea; kuburi79@icf.re.kr (Y.Y.); m930916@icf.re.kr (M.-K.P.); 2 National Institute of Crop Science, Rural Development Administration, Wanju 55365, Republic of Korea; k2h0331@korea.kr; 3 Non-Clinical Evaluation Center, Biomedical Research Institute, Jeonbuk National University Hospital, Jeonjusi 54907, Republic of Korea

In the original publication [1], there were errors in Table 2 and Figure 4D. In Table 2, the primer sequences for SREBP-1c, PPARγ, FAS, C/EBPα, and β-actin were incorrectly listed or partially omitted. The corrected primer sequences are provided below. In addition, Figure 4D previously contained an incorrect immunofluorescence image for UCP-1/DAPI staining. The corrected figure accurately represents UCP-1 expression in differentiated adipocytes treated with DM, WEA, 50EEA, and 70EEA. These corrections do not affect the scientific conclusions of the article. This correction was approved by the Academic Editor, and the original publication has been updated.


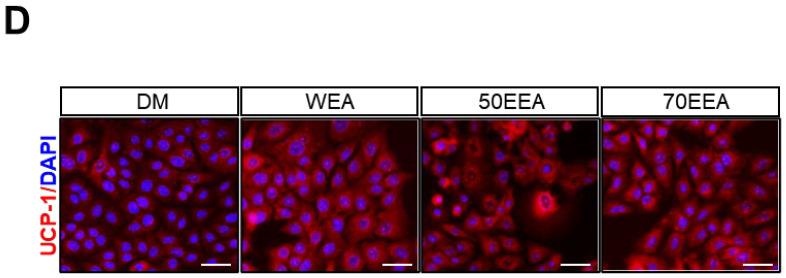

**Table 2:** Real-time PCR primer sequences.

Gene	Sense (5′–3′)	Antisense (5′–3′)
SREBP-1c	CAAGGCCATCGACTACATCCG	CACCACTTCGGGTTTCATGC
PPARγ	CGCTGATGCACTGCCTATGA	AGAGGTCCACAGAGCTGATTCC
FAS	CTCATCCACTCAGGTTCAG	AGGTATGCTCGCTTCTCT
C/EBPα	CGCAAGAGCCGAGATAAAGC	CACGGCTCAGCTGTTCCA
β-actin	AAGACCTCTATGCCAACAC	CTGCTTGCTGATCCACAT
